# The Impact of Perceived Teacher Support on Anti-Immigrant Attitudes from Early to Late Adolescence

**DOI:** 10.1007/s10964-019-00990-8

**Published:** 2019-03-07

**Authors:** Marta Miklikowska, Jochem Thijs, Mikael Hjerm

**Affiliations:** 1 Umeå University, Umeå, Sweden; 2 Utrecht University, Utrecht, The Netherlands

**Keywords:** Anti-immigrant attitudes, Teacher support, Social trust, School context, Adolescence, Attachment

## Abstract

Although research has shown that school context has consequences for intergroup attitudes, few studies have examined the role of teacher qualities, such as teacher support. In addition, previous research has paid limited attention to the mechanisms that could help to explain teacher effects. This 5-wave study (2010–2015) examined the effects of perceived teacher support on the anti-immigrant attitudes of Swedish majority youth (*N* = 671, *M*_*age*_ = 13.41, 50.2% girls, 34 classrooms). It also tested whether social trust would mediate these effects. The results of multilevel analyses showed that perceived teacher support was associated with less prejudice at all levels of analysis. At the within-person level, fluctuations in teacher support were related to fluctuations in youth prejudice: in years when, on average, adolescents perceived their teachers as more supportive, they reported lower prejudice. At the between-person level, adolescents who perceived their teachers as more supportive compared to their peers reported lower prejudice. Similarly, classrooms where students shared an experience of teacher support were lower in prejudice than classrooms with weaker teacher support. The results also showed that social trust explained teacher effects: adolescents who experienced their teachers as more supportive displayed higher levels of trust and, in turn, lower levels of prejudice than youth with less supportive teachers. These findings suggest that teachers can counteract the development of prejudice and facilitate social trust in adolescents by being supportive of them.

## Introduction

Adolescence is a crucial period for ethnic identity development, for both ethnic minority and majority youth (French et al. 2006; Meus 2011; Umaña-Taylor et al. 2014). Apart from exploring the meaning of their group membership, this development involves reflection on the position of their own group relative to that of others, with potentially important implications for intergroup relations and prejudice (Verkuyten 2018). During adolescence, individuals have to balance their concerns about group identity with their moral considerations and growing understanding of egalitarian principles (Rutland and Killen 2015). Such balancing can have different outcomes. For example, two large-scale longitudinal studies found that native majority adolescents become more accepting of immigrant rights over time (Miklikowska 2018; van Zalk and Kerr 2014)—which was explained in terms of their increased understanding of moral principles. Other studies suggest that older compared to younger majority adolescents are more critical and less tolerant of deviating outgroup practices (Gieling et al. 2010), and more likely to condone the exclusion of racial others for non-race related reasons (Killen et al. 2010). Such diverging findings are consistent with Raabe and Beelmann’s (2011) meta-analysis on the development of ethnic, national, and racial prejudice. Although those authors did not find evidence for an overall change in prejudice from early to late adolescence, they reported considerable developmental heterogeneity (with some individuals becoming more and others less prejudiced), suggesting a big role of social contexts in shaping youth attitudes (Raabe and Beelmann 2011). The present study investigated whether the school context, and in particular perceived emotional support and fairness by teachers, is an important source of this variation.

Although research has shown that school context has consequences for intergroup attitudes, previous studies typically looked at the skills taught in school (Hjerm et al. 2017), multicultural content of teaching (Schachner 2017), or school interethnic contact (Thijs and Verkuyten 2014). Very few studies to date have examined how the perceived qualities of the teacher, such as teacher support, can affect the group attitudes and intergroup relations of their students. Also, to the best of our knowledge this research has not focused on adolescents but rather on preadolescent children and paid limited attention to the mechanisms explaining the effects of teachers (Geerlings et al. 2017; Serdiouk et al. 2019). Yet, based on attachment theory (Ainsworth 1989; Bowlby 1982) it can be expected that also adolescents who experience support and care from their teachers become more open to diversity and ethnic others and that this effect might be explained by an increased sense of security and social trust. To test this expectation, data from a large-scale longitudinal study were used. The study measured anti-immigrant attitudes in native Swedish adolescents over five annual waves. The Swedish school system includes nine years of compulsory schooling, where students are exposed to a few teachers, and three years of non-compulsory upper secondary school, where the number of teachers increase.

Sweden is a country of immigration where approximately every fourth person has at least one parent born abroad. The time during the data collection was a period of stable and generous immigration and integration policies that can be exemplified by the number one rank on the Migration and Integration Policy Index in 2015 (MIPEX 2019). At the start of the fieldwork 60 percent of the immigrant population where Swedish citizens and the majority had lived in the country for more than ten years. Although the levels of prejudice have been comparatively low in Sweden, the 2015 asylum crisis and the rise of the radical right have contributed to less generous immigration policies. Such policy changes were not in effect at the time of the data collection but the political discourse was changing in relation to the rise of the radical right (Sweden Democrats) that attracted 12.9% votes in 2014 elections. Even though the party had little influence on the politics during the time period, it did contribute to making immigration issues more salient on the political agenda.

### The Importance of Teacher Support

In the present study the role of teacher support was examined from an extended attachment perspective (Ainsworth 1989; Bowlby 1982; Roorda et al. 2011). Attachment theory states that the bonds that children form with their primary caregivers have life-long importance for the ways they deal with challenges (Ainsworth 1989; Bowlby 1982). Securely attached children can use secure bonds as a “safe haven” to return to in times of stress but also as a “secure base” from which to confidently explore their social worlds. Children also form internal working models of the emotional availability and responsivity of attachment figures, which, in the case of secure attachment allows children to maintain a sense of emotional security when the attachment figure is not present (Bretherton and Munholland 1999). In addition, by satisfying children’s psychological needs, secure attachment frees children from self-preoccupation and, thus, enables them to attend to the needs and perspectives of others (Miklikowska et al. 2011; Sroufe 2005).

Although attachment theory predominantly focuses on child-caregiver relationships, it also acknowledges the importance of other affectional bonds that can be formed throughout development and provide individuals with emotional security and confidence. In her seminal paper “Attachment beyond Infancy”, Ainsworth (1989) suggested several candidates who could function as such surrogate or secondary attachment figures including “an especially perceptive and understanding teacher” (p. 711). Research in primary schools has provided evidence for this by showing that emotionally supportive relations with teachers are important for children, especially for those who are victimized (Little and Kobak 2003) or have behavioral or emotional problems (Baker 2006; Thijs and Fleischmann 2015).

Research on adolescents’ support from teachers has typically not been conducted from an extended attachment perspective. Compared to primary school children, students in middle or secondary school have more teachers, and therefore their perceptions of teacher support have usually been measured by asking them global questions about teachers in general (e.g., Herrero Romero et al. 2018; but see Ryan and Patrick 2001, for an exception). It could be argued that the exposure to more teachers makes individual teachers less important as attachment figures for adolescents. However, research has shown that individuals aggregate their experiences across specific attachment figures within the same domain (e.g., mother, father) leading to global domain-specific attachment orientations (e.g., familial orientation) (Sibley and Overall 2008). Although such aggregation has not been examined in the case of student-teacher relationships, student’s perceptions of different teachers appear to be positively related (Weyns et al. 2017). Thus, it makes sense to study adolescents’ generalized perceptions of support provided by teachers.

It might be argued that these generalized perceptions are relatively unimportant for adolescents, as becoming independent of adults and the formation of own identity are important developmental tasks for them (Berndt 1979; Erikson 1968; Meus 2011). However, “autonomy from significant adults, such as parents and teachers, is not a function of detachment from them but rather a function of attachment and closeness to them” (Davis 2003, p. 223), and despite a decline in the degree of perceived teacher support after late childhood (Bokhorst et al. 2010), teachers continue to be important social agents in adolescence. Research has shown that adolescents who perceive support from their teachers are less likely to have depressive symptoms (Joyce and Early 2014) or initiate health-risk behaviors (McNeely and Falci 2004), more likely to increase in prosocial behavior (Obsuth et al. 2017) and school engagement (Weyns et al. 2017), more likely to intervene in case of peer aggression (Mulvey et al. 2018), and more likely to be protected against the negative impact of peer victimization (Davidson and Demaray 2007). Moreover, affective bonds with teachers appear to be even more important for the academic engagement and achievement of secondary as compared to primary school students (Roorda et al. 2011).

### Teacher Support and Prejudice

Stephan et al. (2009) suggested that people may be predisposed to view outgroup members, such as immigrants, as threatening. The way people think about outgroup members has been suggested to be partly determined by attachment experiences because they are associated with how individuals regulate affect and deal with threat (Carnelley and Boag 2019; Mikulincer and Shaver 2001). According to Bowlby (1982) infants have a natural fear and distrust of the unknown that can threaten their emotional security in the presence of strangers. This sense of security can be restored by the perceived emotional availability of an attachment figure. Knowing and feeling that there is someone to fall back on gives children the confidence to approach challenging situations and thereby mitigates the fear and distrust of unfamiliar others. Secure attachment also acts as template for what can be expected in later relationships, including a sense that other people can be trusted. Moreover, secure attachment has been suggested to facilitate development of a healthy sense of self-worth that, in turn, leads to embracement of less defensive, more tolerant worldviews because it decreases the perception of threat to the self-worth and anxiety associated with it (Hart et al. 2005).

Research offers some support to these claims. In line with attachment theory, Mikunlincer and Shaver (2001) showed that university students with insecure attachment styles reported less positive outgroup evaluations and that the situational activation of relational security (via a secure base priming procedure) improved the evaluations of outgroups by diminishing the experience of outgroup threat. Since Mikunlincer and Shaver’s paper (2001), a small but growing body of research has further supported the notion that attachment security diminishes prejudice toward different types of outgroups while demonstrating the importance of theoretically relevant mediators such as trust, openness to social exploration, and empathy (for a review, see Carnelley and Boag 2019). For instance, experimental research has shown that priming of the romantic relationship attachment style had effects on prejudice and discriminatory behaviour of British majority students (Boag and Carnelly 2016). Security- (versus neutral-) primed tudents reported more positive attitudes toward Muslims and sat closer to a Muslim student they were interacting with. Similarly, Goedert and colleagues (2019) showed that fearful attachment was positively related to more unwelcoming acculturation orientations among Luxembourgish majority adults. Concerning adolescents, Miklikowska and Hurme (2011) reported negative correlations between perceived parental support and youth intolerance (mediated by empathy) and Miklikowska (2016) showed that the perceived quality of relationship with parents moderated the effects of parental prejudice on adolescents’ attitudes toward immigrants (i.e., lower parent-adolescent attitudinal congruence for youth who perceived their parents as less supportive).

Most of previous research has studied adults and there are few studies that have examined how teachers, as secondary attachment figures, can affect the ethnic outgroup attitudes of their students. The exceptions involve recent research in primary school. Serdiouk and colleagues (2019) found that emotional support from teachers was associated with a stronger preference for cross-ethnic friendships among both minority and majority boys in grade 5. They did not obtain this effect for girls or students in grade 1 or grade 3, but it is important to note that teacher support was assessed at the classroom level by independent observations. By contrast, Geerlings et al. (2017) focused on children’s subjective experiences of their teachers. They conducted three studies among native Dutch majority students (grade 4–6) in which they predicted children’s ethnic outgroup evaluations from their perceptions of the relationship with their teacher. Children who perceived this relationship as close and supportive were more positive toward low-status ethnic minority groups, and this effect was independent of their perceptions of, respectively, peer acceptance (Study 1), the child-parent relationship (Study 2), and the multicultural norms of their teacher (Studies 2–3). It was shown to be mediated by a stronger internal motivation to be open to cultural others (Study 3). Additionally, Gniewosz and Noack (2008) showed negative correlations between individual perceptions of teachers’ fairness and intolerant attitudes towards immigrants among early adolescents. These studies are consistent with the extended attachment perspective and indicate that the experience of teacher support has a unique relevance for the evaluations of outgroup others.

### The Role of Trust

The present study examined whether adolescents’ perceptions of teacher support would improve their anti-immigrant attitudes via the promotion of social trust. Social trust involves the belief that people are generally fair and trustworthy, and due to their “widening world of social experiences” and “growth in socio-cognitive competence” it is particularly relevant to study this belief in adolescence (Flanagan and Stout 2010, p. 749). On the one hand, secure attachment implies trust in important others (Ainsworth 1989; Bowlby 1982; Mikulincer and Shaver 2007) and this contributes to generalized expectations of trustworthiness (Glanville and Paxton 2007). On the other hand, a negative relation between trust and intergroup prejudice can be anticipated. Although, social trust has been considered as an outcome rather than as a predictor of diversity and intergroup relations (Sturgis et al. 2011), it implies an expectation that strangers, including people from other cultural backgrounds, are trustworthy. General positive beliefs about people make individuals less suspicious of immigrants who could potentially take advantage of natives or threaten their cultural hegemony. Indeed, social trust has been suggested to make people want to work together for the common good by promoting cooperative social relations (Uslaner 2002; Rothstein and Uslaner 2005).

There is convincing evidence for a link between social trust and attitudes towards immigrants. For instance, two large-scale cross-national studies in 16 and 21 European countries found that people with higher social trust were more positively inclined toward immigrants, also when many different other predictors of prejudice, such as national unemployment levels, were controlled for (Herreros and Criado 2009; Van Linden et al. 2017). These results suggest that social trust leads immigrants to be included in the radius of trusted others and, as a consequence, the benign effects of generalized trust apply to them as well.

To date, there is only limited evidence that teacher support can promote social trust. Flanagan and Stout (2010) found that social trust diminished in adolescence, consistent with their reasoning that “the wider and more diverse social experiences of older adolescents should mean that they are, on average, less naïve and positive than early adolescents in their judgments about people” (p.750). They also found that adolescents’ social trust was boosted by a positive school climate. More specifically, a recent study showed that adolescents’ perceptions of having caring and responsive teachers was positively related to their social trust (Lundberg and Abdelzadeh 2018). However, this study included teacher support only at one time point (in late adolescence). Thus, the result could reflect a positive impact of teacher support that took place earlier in adolescence. In addition, the study did not account for the nested structure of the data. Thus, the exact effects of teacher support are still unclear, because within- and between-person processes might differ (Curran and Bauer 2011), and because the experiences shared with fellow classmates might have a different impact on trust and attitudes towards outgroups due to social comparison processes (Marsh et al. 2008). The current study could overcome these limitations because it followed a sample of 13–14-year-olds until they were 17–18 years of age. In doing so, it could also separate within- and between-person as well as between-classroom processes, and thereby specify the exact effects of teacher support on youth social trust and attitudes towards outgroups.

## Current Study

The goal of this 5-wave longitudinal study was to make a unique contribution to the literature by examining the effects of perceived teacher support on the development of anti-immigrant attitudes of native majority adolescents. Based on attachment theory (Ainsworth 1989; Bowlby 1982) and consistent with prior research on outgroup attitudes in children (Geerlings et al. 2017) and university students (Mikulincer and Shaver 2001, 2007), it was expected that perceived support would predict development of anti-immigrant attitudes in adolescence and that its effects would be mediated by youth social trust.

A multilevel approach was used that distinguished between within-person changes (i.e., average year-to-year fluctuations; Level 1) as well as between-person (Level 2) and between-classroom differences (i.e., between all native students in each classroom; Level 3). Hence, it was possible to examine whether fluctuations in perceived teacher support were associated with fluctuations in anti-immigrant attitudes (Level 1), but also to investigate whether teacher support predicted differences in the level and rate of change in anti-immigrant attitudes (via differences in social trust) both at the student (Level 2) and the classroom level (Level 3). Distinguishing between the individual student and the classroom level is important as the experience students have with teachers are sometimes comparable to those of other adolescents or their classmates but also unique (due to teachers’ differential behaviors toward students, see for example Ahn et al. 2018). These shared and unique experiences could have a different impact on trust and anti-immigrant attitudes because students might rely on social comparison. For example, the literature on the so-called big-fish-little-pond phenomenon has shown that, although academic achievement has a positive effect on children’s academic self-concept, this self-concept is weaker in classes with high average achievement (Marsh et al. 2008). This could suggest that it is only adolescents’ unique experiences of teacher support (relative to other adolescents’ or their classmates), that increase trust and reduce anti-immigrant attitudes and that the experiences they share with their classmates do not matter so much.

In testing the hypotheses, the direct effects of youth and classroom socio-economic status, classroom diversity, and their interactions with teacher support were controlled for. These factors have been shown to have consequences for youth intergroup attitudes (Miklikowska 2017; Thijs and Verkuyten 2014), trust (Stolle et al. 2008), and teacher engagement at school (Demerouti et al. 2001).

## Method

### Participants and Procedure

The 5-wave data were collected between 2010 and 2015 in the Swedish seventh largest city of 137,000 inhabitants. The city resembles the national average on income (Örebro city/Sweden 30kEuro/31.8kEuro), unemployment (Örebro city/ Sweden 7.2/7.0), and ethnic diversity (Örebro city/Sweden 17.6/18.6%) (Statistiska Centralbyrån 2016). The data were collected in 10 schools selected from a range of neighborhoods with different ethnic and social backgrounds. Every class received yearly a small payment of 100 EUR for participation. Only 3.8% of parents refused their children’s participation in the study. The data were collected during school hours and trained research assistants administered questionnaires. Adolescents received information about the confidentiality of their answers, voluntary character of their participation, and the types of questions in the questionnaires. This study uses the data that are a part of a larger study on youth political development. The study was ethically approved by the Regional Ethical Review Board in Uppsala (Dnr, 2010/115).

Adolescents that formed the initial sample (*N* = 946; *M*_*age*_ = 13.41; *SD* = 0.53; 50.7% girls) had varying perceived family finances and ethnic background (71.5% Swedish majority youth, 27.1% youth with an immigrant background, i.e., with at least one parent born outside of the Nordic countries, 16% had both parents born outside of Europe). Adolescents were nested in 38 classrooms that were stable between T1 and T3. The stability theoretically includes teachers as the same teachers tend to teach the same classes during seventh to ninth grade. However, there was no information on teachers in the data. Thus, it is uncertain to what extent adolescents were exposed to the same or different teachers during those three years due to teacher replacement from those changing jobs, schools, or retiring. At T4 adolescents transitioned from middle schools to upper secondary schools, which means that they changed schools, classrooms, teachers, and classmates.

Given that the dependent variable captured attitudes towards immigrants and attitudes of Swedish adolescents with immigrant background have been shown to be more positive and follow a different pattern of change than attitudes of Swedish majority youth (Lundberg and Abdelzadeh 2017), participants with an immigrant background (i.e., with at least one parent born outside of the Nordic countries; N = 256, 2 classrooms) were excluded from the analyses. They were present in the construction of classroom measures of teacher support, diversity, and average classroom family finances to capture a shared experience of teacher support in the classroom and to capture an objective assessment of classroom diversity and average classroom family finances. In addition, after removing youth with immigrant background, two classrooms with less than four participants (initially composed of predominantly immigrant youth) were excluded in order to eliminate possible bias for aggregated class and individual-level variables. The final sample consisted of *N* = 671 adolescents, *M*_*age*_ = 13.41, 50.2% girls, nested in 34 classrooms. The data were organized by age: youth 13–14 years old at T1 (*M* = 13.41, *SD* = 0.52), 14–15 years old at T2 (*M* = 14.27, *SD* = 0.44), and 15–16 at T3 (*M* = 15.35, *SD* = 0.48), 16–17 at T4 (*M* = 16.30, *SD* = 0.47) and 17–18 at T5 (*M* = 17.33, *SD* = 0.48).

To test whether the dropout of adolescents from T1 to T5 (*N* = 214) was related to the background and the study variables, logistic regression analyses were performed testing whether attrition (dropout = 0, retention = 1) was predicted by the background (gender, family structure [intact vs. not intact], perceived family finances) or the study variables. The results showed that dropout was not significantly related to background variables with exception of perceived family finances. Although adolescents with higher perceived family finances were more likely to participate at T5 than adolescents with lower perceived family finances (χ^2^ (1, *N* = 613) = 7.69, *p* < 0.01), a low value of Nagelkerke R^2^ = 0.02 indicated that this would have small chance of affecting the analyses (Borooah 2001). Attrition was significantly related to adolescents’ social trust and perceived teacher support (χ^2^ (1, *N* = 633) = 5.42, *p* < 0.05 and χ^2^ (1, *N* = 632) = 5.84, *p* < 0.05, respectively). Youth with higher social trust and who perceived their teachers as supportive were more likely to participate at T5 but low values of Nagelkerke R^2^ = 0.01, suggested minor issues. Inspection of the data for missing values showed that the average proportion of missing data for all study variables was 18% To account for the missing data, the Full Information Maximum Likelihood (FIML) was used. FIML has been shown to provide more reliable standard errors than mean imputation or listwise or pairwise deletion (Little and Rubin 2002).

### Measures

#### Anti-immigrant Attitudes

At all time points adolescents reported on their attitudes toward immigrants by rating three items on a 4-point Likert scale (1 = Don’t agree at all to 4 = Agree completely): “Immigrants often come here just to take advantage of welfare in Sweden”, “It happens too often that immigrants have customs and traditions that not fit into Swedish society”,” Immigrants often take jobs from people who are born in Sweden”. The mean of items was used to construct the scale score. The average scores ranged from 1 to 4 (*M* = 2.29, *SD* = 0.57). These items have commonly been used before to tap anti-immigrant sentiment (ESS 2002–2018; Miklikowska 2018; Van Zalk and Kerr 2014). The internal reliabilities for the items in this study were good 0.76, 0.79, 0.79, 0.81, and 0.84. These estimates are similar to those reported in previous studies. These studies have also shown the scale to have convergent, predictive, and discriminant validity.

#### Perceived Teacher Support

At all time points adolescents reported on their perceived teacher support by rating five items on a 4-point Likert scale (1 = Absolutely agree at all to 4 = Absolutely disagree): “Most of my teachers treat me fairly” (item reversed), “Most of my teachers listen to what I have to say” (item reversed), “There are scarcely any teachers who I can talk with if I have problems with something at school”, “Most teachers are keen that their students feel good” (item reversed), “There are scarcely any teachers who praise me when I do a good job”. The mean of items was used to construct the scale score. The average scores ranged from 1 to 4 (*M* = 3.07, *SD* = 0.45). The internal reliabilities for the items in this study were good 0.75, 0.78, 0.80, 0.79, 0.84, for T1-T5, respectively. In addition, confirmatory factor analysis showed that the items loaded on one factor, with an average loading of 0.74 across all time points.

#### Social Trust

At all time points adolescents reported on their social trust by rating two items on a 5-point Likert scale (1 “Doesn’t apply at all” to 5 “Applies perfectly): “Most people can be trusted”, “Most people are fair, and don’t try to exploit you”. The mean of items was used to construct the scale score. The average scores ranged from 1 to 5 (*M* = 3.14, *SD* = 0.65). The internal reliabilities for the items in this study were good 0.78, 0.75, 0.79, 0.82, 0.84, for T1-T5, respectively. Spearman-Brown coefficients were also good: 0.79, 0.75, 0.79, 0.82, 0.84. In addition, confirmatory factor analysis showed that the items loaded on one factor, with an average loading of 0.89 across all time points.

#### Perceived Family Finances

At all time points adolescents reported on their perceived family finances by rating 1 item “What are your family finances like?” on a 4-point scale ranging from 1 = My parents always complain that they don’t have enough money to 4 = My parents never complain about being short of money. The average scores ranged from 1 to 4 (*M* = 3.06, *SD* = 0.63). Family finances was computed at the individual level but also averaged at the classroom level to capture differences in classrooms’ socio-economic situation. A measure of perceived family finances was used given that using a more objective measure reported by parents would reduce the sample size by almost 50 percent and limit the available measurement points from five to three. In addition, it has been shown that perceived familial SES, of which family finances is a constituent part, can be a better predictor of perceived health outcomes than objective SES as it can be seen as a form of identity (Goodman et al. 2007). Although health outcomes were not the focus of this study, a perception was and thus it could be assumed that subjective family finances at least would not be worse than an objective measure.

#### Classroom Diversity

Classroom diversity was operationalized as a proportion of adolescents with an immigrant background (i.e., with at least one parent born outside of the Nordic countries). Classroom diversity scores ranged from 0 to 0.56 (*M* = 0.20, *SD* = 0.14) at T1, from 0 to 0.59 (*M* = 0.19, *SD* = 0.14) at T2, and from 0 to 0.57 (*M* = 0.19, *SD* = 0.14) at T3, respectively. At T4 adolescents transitioned from middle school to upper secondary school (i.e., they changed schools and were assigned to new and different classrooms). These classrooms were largely comprised of youth outside of the current sample and thus there is not enough information to compute diversity scores for T4 and T5. This also includes the school level as the individuals in the dataset comprise a too small proportion of the upper secondary schools to be able to make such calculation. Nor is such information available elsewhere. The total average classroom diversity at T1, T2, and T3 was treated as a proxy for adolescents’ experience of classroom diversity. The classroom diversity was very stable over time (T1-T3) as it was only affected by adolescents moving to or from the school. Thus, the level three classroom diversity variable is a non-time-varying covariate. The diversity scores for eleven adolescents who changed classrooms between T1-T3 were treated as missing values. The average classroom diversity scores ranged from 0 to 0.56 (*M* = 0.20, *SD* = 0.14).

### Preliminary Analyses

Correlations between the study variables can be found in Table 1. Adolescents’ anti-immigrant attitudes were consistently related to perceived teacher support and social trust. Perceived teacher support was consistently related to youth social trust. In addition, adolescents’ anti-immigrant attitudes were not significantly related to the perceived family finances but related to classroom diversity (*r* = −19, *r=* −0.09 at T2 and T3, respectively). Teacher support was significantly related to the perceived family finances (*r=* 0.17, *r* = 0.13, r = 0.19, and r = 0.14, *p* < 0.001 at T2-T5, respectively) and classroom diversity (*r=* 0.10, *p* < 0.05 at T3). Social trust was significantly related to the perceived family finances (*r* = 0.09, *r=* 0.17, *r* = 0.19, r = 0.15, and r = 0.23, *p* < 0.05 at T1-T5, respectively) but not to classroom diversity.

**Table 1:** Correlations between the study variables

Variables	1.	2.	3.	4.	5.	6.	7.	8.	9.	10.	11.	12.	13.	14.	15.
1. Prejudice T1	—														
2. Prejudice T2	0.444**	—													
3. Prejudice T3	0.420**	0.579**	—												
4. Prejudice T4	0.372**	0.446**	0.650**	—											
5. Prejudice T5	0.234**	0.402**	0.534**	0.673**	—										
6. Support T1	−0.128**	−0.185**	−0.175**	−0.138*	−0.102*	—									
7. Support T2	−0.080	−0.147**	−0.164**	−0.119*	−0.133*	0.545**	—								
8. Support T3	−0.073	−0.134*	−0.170**	−0.128*	−0.167**	0.412**	0.571**	—							
9. Support T4	−0.021	−0.121*	−0.180**	−0.227**	−0.157**	0.316**	0.378**	0.477**	—						
10. Support T5	−0.028	−0.103*	−0.094*	−0.078	−0.105*	0.284**	0.319**	0.417*	0.472**	—					
11. Trust T1	−0.083*	−0.094*	−0.114*	−0.125*	−0.005	0.363**	0.233**	0.214**	0.110*	0.073	—				
12. Trust T2	−0.080	−0.135**	−0.219**	−0.200**	−0.120*	0.284**	0.381**	0.256**	0.281**	0.184**	0.459**	—			
13. Trust T3	−0.132*	−0.127*	−0.198**	−0.171**	−0.191**	0.252**	0.312**	0.361**	0.212**	0.210**	0.389**	0.466**	—		
14. Trust T4	−0.135*	−0.153*	−0.272**	−0.303**	−0.173**	0.193**	0.318**	0.313**	0.322**	0.183**	0.320**	0.497**	0.561**	—	
15. Trust T5	−0.053	−0.129*	−0.208**	−0.200	−0.274**	0.138*	0.170**	0.338**	0.240**	.259**	0.234**	0.337**	0.491**	0.522**	—

*p < 0.0N5, **p < 0.01

To assess mean-level changes in adolescents’ attitudes, perceived teacher support, and social trust, repeated measures analyses of variance were performed, with measurement time as a within-subject variable and adolescents’ outcomes as dependent variables. Adolescents’ attitudes showed significant changes between T1 and T5 *F*(4, 316) = 7.00, η_p_^2^ = 0.022, *p* < 0.001. Pairwise comparisons with Bonferroni correction showed a significant mean-level decrease between T4 and T5. Adolescents’ perceived teacher support showed significant changes between T1 and T5 *F*(4, 342) = 19.54, η_p_^2^ = 0.054, *p* < 0.001. Pairwise comparisons with Bonferroni correction showed a significant mean-level increase between T3 and T4, that is when adolescents changed schools and teachers. Adolescents’ trust showed significant changes between T1 and T5 *F*(4, 340) = 22.14, η_p_^2^ = 0.061, *p* < 0.001. Pairwise comparisons with Bonferroni correction showed a significant mean-level increase between T2 and T3.

### Strategy of Analyses

Mplus 8 (Muthén and Muthén 1998–2017) and multilevel analyses were used. To test whether perceived teacher support would predict within- and between-person changes in adolescents’ attitudes multilevel models were built with time nested within adolescents, nested within classrooms. All time-constant predictors at Level 2 and Level 3 were grand-mean centered (i.e., based on the sample average of the means from 5 time points) and time-varying predictors at Level 1 were person-mean centered (i.e., based on the individual average of the means from 5 time points) to create level-specific variables as partitioning is not yet available for independent variables (Muthén and Muthén 1998–2017). First, baseline models were specified to assess average developmental change (Model 1 and Model 2). These models included: a random intercept that split the variance in youth attitudes into within-person (i.e., fluctuations of the same adolescent over time), between-person (i.e., differences in levels between adolescents), and between-classroom levels (Model 1), a fixed linear slope and quadratic that represented the average change of all adolescents’ attitudes (Model 2), given that previous studies showed a non-linear development of anti-immigrant attitudes in adolescence (Miklikowska 2017). In the next step, a predictor of the fixed intercept was modeled, i.e., perceived teacher support at the within-person level (Model 3). Next, a random slope that represented differences in change over time that might vary between persons or classrooms was modeled (Model 4). Finally, teacher support was modeled as a predictor of the random intercept and slope at the between-person level and of a random intercept at the between-classroom level (Model 5). Additional analyses were carried out to examine that the within- and between-person as well as between-classroom effects of teacher support have not changed when controlling for adolescents’ perceived family finances and classroom diversity.

To examine whether adolescents’ social trust would mediate the within- and between-person effects of perceived teacher support, mediation model was specified. It included direct paths from teacher support to the level of adolescents’ attitudes and social trust as well as a direct path from youth social trust to their attitudes. Significance of the indirect effect was tested by bootstrapping confidence intervals (Preacher and Hayes 2008). All nested models were evaluated using deviance test (Δ-2LL) and AIC criterion. The model with lower deviance and AIC, compared to the null model, is considered to be the better-fitting model (Kreft and de Leuw 1998).

## Results

### Does Perceived Teacher Support Predict Within- and Between-person Changes in Adolescents’ Attitudes?

To test whether perceived teacher support would predict changes in youth anti-immigrant attitudes, first a random intercept model was built that splits the variance into within and between adolescents, as well as between classrooms (Model 1). The interclass correlations (ICC) from this unconditional model showed that 53.5% of variance in attitudes was found within adolescents, 39.6% of variance was found between adolescents and 6.9% of variance was found between classrooms. Adding fixed linear and quadratic slopes (Model 2) significantly improved model fit (Δ-2LL = 32.58(2), *p* = 0.001; ΔAIC = 28.57) and reduced unexplained within-person variance. The average linear slope in youth attitudes was significant (B = 0.125, *p* < 0.001, 95%CI [0.082, 0.167]) as well as the average quadratic slope (B = −0.035, *p* < 0.001, 95%CI [−0.045, −0.024]), indicating a linear increase followed by a decrease in anti-immigrant attitudes. Controlling for the average linear and quadratic changes, in the next step, teacher support was entered as a predictor of within-person fluctuations of prejudice (Model 3). This significantly improved model fit (Δ-2LL = 4.68 (1), *p* < 0.05; ΔAIC = 2.67) and reduced unexplained within-person variance. The results showed that within-person fluctuations in adolescents’ attitudes were significantly related to fluctuations in perceived teacher support (B = −0.064*, p* < 0.05, 95%CI [−0.112, −0.015]).

Adding a random linear slope at the between-person level (Model 4) significantly improved model fit (Δ-2LL = 89.46 (1), *p* < 0.001; ΔAIC = 87.46) and reduced unexplained between-person variance. There was a significant variation around the linear slope at the between-person level (σ² = 0.027, *p* < 0.001, 95%CI [0.021, 0.033]), indicating that there were differences between adolescents in their rate of change. The slope variance at the classroom level was not significant (σ² = 0.002, *p* = ns., 95%CI [0.004, 0.003]), indicating that there were no differences between classrooms in the rate of change, nor the correlation between level and slope (B = 0.003, *p* = ns., 95%CI [−0.001, 0.007]) and thus they were constrained to zero. Although significant at the within-person level, adding a random quadratic slope at the between-person and between-classroom level did not improve model fit, indicating that there were no differences between individuals and classrooms in their rate of change.

In the next step, teacher support was modeled as a predictor of the random intercept and slope at the between-person and a random intercept at the between-classroom level (Model 5). This further improved model fit (Δ-2LL = 30.66 (3), *p* < 0.001; ΔAIC = 24.67) and reduced unexplained variance at the between-person and between-classroom level (Model 4). The results showed that, at the between-person level, teacher support significantly predicted the level of youths’ anti-immigrant attitudes (B = −0.15, *p* < 0.01, 95%CI [−0.252, −0.055]) but not the slope of youth prejudice (B = −0.039, *p* = 0.075, 95%CI [−0.076, −0.003]). Adolescents who perceived their teachers as more supportive displayed lower levels of prejudice than youth with less supportive teachers. Every one unit increase in teacher support was associated with a 0.15 decrease in youth anti-immigrant attitudes.

Similarly, at the between-classroom level, teacher support significantly predicted the level of anti-immigrant attitudes (B = −0.501, *p* < 0.05, 95%CI [−0.864, −0.137]), showing that adolescents attending classrooms where students perceived their teachers as supportive displayed lower levels of prejudice that youth attending classrooms where teachers were perceived as less supportive. Every one unit increase in teacher support was associated with a 0.50 decrease in classroom anti-immigrant attitudes. Comparable results were obtained when immigrant youth were excluded from the class averages of perceived teacher support. All model fit indices and parameter estimates are presented in Table 2.

**Table 2:** Models to account for change in adolescents anti-immigrant attitudes

Model	Fit LL (df)	Change 2LL (df)	Level 1 Within Person (B)	Level 2 Between Person (B)	Level 3 Between classroom (B)	Variance Level 1	Variance Level 2	Variance Level 3
Model 1 Unconditional	−2616.14 (4) AIC = 5240.27	—	—	—	—	0.294***	0.218***	0.038**
Model 2 Fixed Linear and Quadratic Slope (Level 1)	−2599.85 (6)	32.14 (2)	B_lin_ = 0.125	—	—	0.290***	0.218***	0.037**
	AIC = 5211.70	*p* < 0.001	*p* = 0.001					
			B_qdr_ = −0.035					
			*p* < 0.001					
Model 3 Predictor: Teacher Support (Level 1)	−2597.51 (7)	4.68 (1)	B_lin_ = 0.125	—	—	0.289***	0.219***	0.038**
	AIC = 5209.03	*p* < 0.05	*p* < 0.001					
			B_qdr_ = −0.035					
			*p* < 0.001					
			B = −0.064					
			*p* < 0.05					
Model 4 Random Linear Slope (Level 2)	−2552.78 (8)	89.46 (1)	B_qdr_ = −0.006	B_Level*Slope_ = −0.034	—	0.228***	0.293***	0.034**
	AIC = 5121.57	*p* < 0.001	*p* < 0.05	*p* < 0.001				
			B = −0.049					
			*p* = ns.					
Model 5 Predictor:	−2537.45 (11)	30.66 (3)	B_qdr_ = −0.005	B_Level*Slope_ = −0.034	B_Level_ = −0.501	0.228***	0.289***	0.023*
Teacher Support (Level 2)	AIC = 5096.90	*p* < 0.001	*p* < 0.05	*p* < 0.001	*p* < 0.05			
Teacher Support (Level 3)			B = −0.070	B_Level_ = −0.15				
				*p* < 0.01				
			*p* < 0.05					
				B_Slope_ = −0.04				
				*p* = ns.				

*p < 0.05, **p < 0.01, ***p < 0.001

Additional analyses showed that the within-person effects of teacher support did not change substantially (B = −0.066, *p* < 0.05) when controlling for perceived family income and its interaction with teacher support (B = −0.024, *p* = ns. and B = 0.001, *p* = ns., respectively). When controlling for classroom average perceived family finances and its interaction with teacher support, the between-classroom effect of teacher support was significant (B = −0.47, *p* < 0.05). The effects of classroom average perceived family finances and the effect of the interaction between classroom average perceived family finances and teacher support were not significant (B = −0.154, *p* = ns. and B = 0.586, *p* = ns., respectively). Similarly, when controlling for classroom diversity and the interaction between classroom diversity and teacher support, the effect of teacher support was significant (B = −0.44, *p* < 0.05). The effect of classroom diversity was also significant (B = −0.495, *p* < 0.05) but the effect of the interaction between classroom diversity and teacher support was not (B = 0.69, *p* = ns.).

### Does Social Trust Account for the Effects of Perceived Teacher Support?

To test whether adolescents’ social trust would mediate the within- and between-person effects of teacher support, a mediation model was specified. It included direct paths from teacher support to the level of adolescents’ attitudes and social trust as well as a direct path from youth social trust to their attitudes. At the within-person level, the indirect effect of teacher support on youth attitudes via their social trust was significant (B = −0.014, *p* < 0.001; 95%CI [−0.021, −0.007]). The direct effect of teacher support on youth social trust was significant (B = 0.206, *p* < 0.001; 95%CI [0.158, 0.253]) as well as a direct effect of social trust on the youth attitudes (B = −0.067, *p* < 0.001; 95%CI [−0.098, −0.037]). The direct effect of teacher support on youth attitudes was no longer significant (B = −0.050, *p* = ns.; 95%CI [−0.100, 0.001]). At the between-person level, the indirect effect of teacher support on the level of youth attitudes via their social trust was significant (B = −0.117 *p* < 0.001; 95%CI [−0.161, −0.072]). The direct effect of teacher support on youth social trust was significant (B = 0.645, *p* < 0.001; 95%CI [0.557, 0.733]) as well as a direct effect of social trust on the level of youth attitudes (B = −0.181, *p* < 0.001; 95%CI [−0.244, −0.117]). After accounting for indirect effect via trust, the direct effect of teacher support on youth attitudes was no longer significant (B = −0.031, *p* = ns.; 95%CI [−0.138, 0.077]).

At the between-classroom level, the indirect effect of teacher support on the level of youth attitudes via their social trust was not significant (B = −0.017 *p* = ns.; 95%CI [−0.226, 0.191]). The direct effect of teacher support on youth social trust was significant (B = 0.687, *p* < 0.001; 95%CI [0.362, 1.013]) but the direct effect of social trust on the level of youth attitudes was not (B = −0.025, *p* = ns.; 95%CI [−0.328, 0.278]). The direct effect of teacher support on youth attitudes was significant (B = −0.515, *p* = 0.035; 95%CI [−0.916, −0.113]). Comparable results were obtained when immigrant youth were excluded from the class averages of perceived teacher support. Alternative mediation models were tested to examine whether perceived teacher support would mediate the effects of trust on youth anti-immigrant attitudes. The results showed insignificant indirect effects at all three levels (B = −0.005, *p* = ns.; 95%CI [−0.009, 0.000] and B = −0.027, *p* = ns.; 95%CI [−.055, 0.002] at the within- and between-person level as well as B = −0.021, *p* = ns.; 95%CI [−0.040, −0.027] at the between-classroom level). Together these results suggest that there is stronger evidence for trust as a mediator between perceived teacher support and prejudice than for teacher support as a mediator between trust and prejudice.

## Discussion

This study examined how native adolescents’ attitudes towards immigrants are affected by their perceptions of teacher support. Although there is ample support for the idea that school context is important for students’ ethnic outgroup attitudes via, for example, multicultural teaching (Hjerm et al. 2017; Schachner 2017) or intercultural contact (Thijs and Verkuyten 2014), very few studies have examined the prejudice-reducing potential of teacher qualities, such as teacher support. Moreover, the little research available focused on pre-adolescent students, used cross-sectional data, and paid limited attention to the processes explaining teacher effects (Geerlings et al. 2017; Serdiouk et al. 2019). By contrast, this study used five waves of longitudinal data and a multilevel approach to examine a more nuanced role of perceived teacher support in the development of adolescents’ attitudes towards immigrants. In doing so, it could examine whether fluctuations in perceived teacher support were associated with fluctuations in anti-immigrant attitudes (Level 1), whether teacher support predicted differences in prejudice both at the student (Level 2) and the classroom level (Level 3), and whether social trust would help to explain these effects.

As expected, the results indicated that perceived teacher support was consistently associated with weaker anti-immigrant attitudes at all levels of analysis. At the within-person level, increases in perceived teacher support were related to decreases in youth prejudice, and at the between-person level, youth who generally perceived their teachers as more supportive compared to their peers reported lower levels of anti-immigrant prejudice. Similarly, the average degree of teacher support at the classroom level explained between-classroom differences in anti-immigrant attitudes. Classrooms where students shared an experience of teacher support were lower in prejudice than classrooms with weaker perceived teacher support. Moreover, and consistent with our hypotheses, the effects of perceived teacher support were mediated by adolescents’ social trust at the within- and between-person level. Adolescents who perceived their teachers as more supportive displayed higher levels of trust and, in turn, lower level of prejudice than youth with less supportive teachers.

These results are in line with the extended attachment perspective on the role of teacher support (Ainsworth 1989; Bowlby 1982; Roorda et al. 2011) and the notion that a sense of secure attachment can diminish prejudice toward potentially threatening outgroups (Boag and Carnelley 2016; Carnelley and Boag 2019; Mikulincer and Shaver 2001). They also support the idea that trust is crucial for positive attitudes toward immigrants (Herreros and Criado 2009; Van Linden et al. 2017) and show that supportive teachers are relevant for promoting this trust in adolescents (Lundberg and Abdelzadeh 2018).

Adolescents’ relations with their teachers are not as exclusive and enduring as the bonds with their primary attachment figures (e.g., parents). Still, the extended attachment perspective claims that supportive relations with teachers can provide students with the security to explore their social worlds and approach challenging situations (see Ainsworth 1989). The results of our analyses showed that there were significant fluctuations in adolescents’ perceptions of teacher support, which suggests that youth perceptions of their teachers are malleable and reflect their unique experiences over time. However, there was also considerable between-person variation in perceived teacher support indicating individual differences in adolescents’ experiences of their teachers and, thus, in the possibility to “use” them as secondary attachment figures. The combination of within- and between-person variation indicate that adolescents organize the experiences with their teachers in representations that partly susceptible to change and partly consistent and stable over time (Fraley 2002). This, and the fact that students aggregated their experiences with different teachers into reliable perceptions of teacher support at each wave, further supports the idea of global domain-specific attachment orientations (Sibley and Overall 2008). It seems that students did not only aggregate their experiences with different teachers into reliable perceptions of teacher support at each wave but also showed considerable consistency in these perceptions over the years. At the same time, there were small but systematic differences between classrooms in perceived teacher support suggesting that adolescents’ perceptions of their teachers were partly similar to those of their classmates’ due to shared experiences.

From an attachment perspective, the experience of supportive and secure relations with important others such as teachers should make individuals less afraid of strangers, including members of outgroups (Bowlby 1982; Mikulincer and Shaver 2001), and one of the explanations for this is that such relations promote a sense of social trust (for review see Carnelley and Boag 2019; Lundberg and Abdelzadeh 2018; Glanville and Paxton 2007). Our findings are clearly consistent with this idea and with the conclusion that people’s situational experience of attachment security and their global attachment styles are independent predictors of their outgroup attitudes (Mikulincer and Shaver 2001). Perceived teacher support was found to be consistently related to weaker anti-immigrant attitudes and higher social trust at all levels of analysis and the relation between teacher support and anti-immigrant attitudes was mediated by social trust at the within- and the between-person level.

In particular, at the within-person level, the fluctuations (i.e., increases or decreases) in adolescents’ perceptions of teacher support were indirectly related to fluctuations in their anti-immigrant attitudes via an increase (or decrease) in social trust. In years when, on average, youth perceived their teachers as more supportive than usually, they reported lower prejudice. This is a promising result, as it highlights the plasticity of youth perceptions of teachers so that experiences of supportive teachers may yield relatively fast decreases in prejudice and increases in trust. It also suggests, however, that youth attitudes and trust, once changed, may relapse easily if their perceptions of teacher support change. At the between-person level, adolescents who generally perceived their teachers as more supportive than their peers reported higher levels of trust and, in turn, lower levels of anti-immigrant prejudice. This suggests that adolescents’ prejudice and trust not only co-fluctuate with the increases or decreases in teacher support but also that the adolescents’ individual experiences of teacher support (relative to other adolescents’) matter for their level of youth trust and prejudice. This suggest that social comparison processes (e.g., Marsh et al. 2008) might be relevant here. Moreover, although there was a significant variation in adolescents’ linear change in prejudice at the between-person level, this variation was not predicted by the level of perceived teacher support (relative to other adolescents’). Nevertheless, the between-person trajectories of youth prejudice were at different levels depending on the overall support provided by teachers (i.e., higher for adolescents who perceived their teachers as less supportive).

In addition to these within- and between-person processes, perceived support predicted social trust and anti-immigrant attitudes also at the between-classroom level. Adolescents attending classrooms where all teachers were generally experienced as supportive had higher social trust and lower anti-immigrant prejudice than their counterparts from less-supportive classrooms. This suggests that it is not only adolescents’ unique individual experiences of teacher support (relative to other adolescents’) that matter for their trust and prejudice but that the experiences of teachers shared with their classmates also play a role. Still, the mediation hypothesis was not supported at the classroom level as the between-classroom differences in social trust were unrelated to the between-classroom differences in attitudes toward immigrants. This suggests that, unlike their unique trust beliefs (i.e., those non-shared with classmates), adolescents’ shared beliefs about the trustworthiness of others are irrelevant for the evaluation of immigrant outgroups. A tentative interpretation for this finding could be that adolescents’ trust beliefs have little psychological salience when there is little disagreement with others. However, if these general beliefs stand out relative to those of their classmates, adolescents might be more likely to reflect on them and “apply” them to specific outgroups such as immigrants. Future research could test this interpretation by examining the salience of adolescents’ trust beliefs.

It might be argued that the present findings can also be interpreted from a social learning perspective (Bandura 1977). By observing supportive and positive behaviors from teachers toward themselves, students might learn that it is normative to treat others the same way, and this in turn, could make them more open to immigrant others (Gniewosz and Noack 2008). Although there is something to be said for this interpretation, the extended attachment approach provides a better explanation. To properly evaluate the social learning perspective, it would be necessary to also examine teachers’ various ways of communicating in the classroom, such as, for instance, their normative teachings about diversity (Hjerm et al. 2017; Schachner 2017) and their own attitudes towards immigrants. It is unlikely that students would develop positive attitudes from observing supportive teachers who express negative attitudes. It would be important to also consider the peer group norms as peers are attitudinal role models in adolescence (Miklikowska 2017; Van Zalk and Kerr 2014). Unlike the social learning perspective, the extended attachment perspective focuses on the security that teachers provide to their students, and the impact of this security on students’ outgroup attitudes appears to be independent of teachers’ communications about diversity (Geerlings et al. 2007).

The current research has various strengths. Due to its long-term longitudinal design, this study examined the effects of teacher support over an extended period of time (across adolescence). It also used multilevel analysis and differentiated the within- and between-person effects as well as between-classroom processes. Thus, it captured more nuanced effects of teacher support, where adolescents’ unique, relative, and shared experiences of teachers were tested. Moreover, this study also examined a mechanism behind main effects. Thus, it could explain how support from teachers improved adolescents’ attitudes towards immigrants. Finally, this study focused on adolescence, where the role of teacher qualities has rarely been examined. By doing so, it could show that relationships with secondary attachment figures such as teachers are important for intergroup relations beyond childhood.

Nevertheless, it also has some limitations. First, teacher support was measured as a perception and, thus, can differ from “actual” support. Previous research has shown, for example, relatively low agreement between child perceptions and teacher self-reports (Li et al. 2011). However, it would have been practically difficult for this study to measure the actual support that individual students receive from multiple teachers over the years. Moreover, the theoretical interest of this study was in adolescents’ personal experiences and these might be more influential than the “actual” support. Second, the direction of effects needs more attention. It is possible that students who are prejudiced perceive their teachers as less supportive (Weyns et al. 2017). We tested this possibility with random-intercept cross-lag model and the results showed bi-directional effects (results can be obtained from the authors upon request). Third, the change of schools and teachers between T3 and T4 was accompanied by an increase in perceived support. We do not know if this is a result of teachers being, on average, more supportive at the new schools or if the transition itself brought the change in perceived support (i.e., we could not separate a change in support from a different set of teachers from a long-term support emanating from exposure to the same set of teachers T1-T3). This does not refute the result that a change in perceived support is associated with a change in prejudice. Fourth, it is likely that trust is not the only mechanism that connects teacher support and anti-immigrant attitudes and other studies should examine, for instance, the role of empathy (Carnelley and Boag 2019; Miklikowska and Hurme 2011). Fifth, given that previous research shows that attachment in other contexts, with parents or romantic partners, can also play a role in the formation of intergroup attitudes (Boag and Carnelly 2016; Goedert et al. 2019; Miklikowska 2016; Miklikowska and Hurme 2011), future studies should examine how attachments with various figures interact in producing prejudice. Finally, there was no information on the teachers in the data above the adolescents´ evaluation. Thus, we could not examine if teacher traits had any moderating effects.

## Conclusions

Although research has shown that school context has consequences for intergroup attitudes, few studies have examined the role of teacher qualities and the mechanisms that could explain their effects. This longitudinal study makes a unique contribution to the literature by examining the nuanced effects of perceived teacher support on the development of anti-immigrant attitudes of native majority adolescents and by testing whether these effects were mediated by social trust. The multilevel analyses showed that teacher support was consistently associated with weaker anti-immigrant attitudes at all levels of analyses. Within-person increases in perceived teacher support were related to decreases in prejudice, youth who perceived their teachers as more supportive compared to their peers reported lower levels of anti-immigrant attitudes, and classrooms where students shared an experience of teacher support were lower in prejudice than classrooms with weaker teacher support. The analyses also showed that the effects of teacher support were mediated by social trust at the within- and between-person level. Adolescents who perceived their teachers as more supportive displayed higher trust and, in turn, lower prejudice than youth with less supportive teachers. These findings suggest that teachers can counteract the development of prejudice and facilitate social trust in adolescents by being supportive of them. They also suggest that research on multicultural education or school interethnic contact should include teacher support as a relevant variable to better understand the role of school context for youth intergroup relations, and thereby to inform school-based attempts of changing these relations for the better.
